# Structural Characterization of the Loop at the Alpha-Subunit C-Terminus of the Mixed Lineage Leukemia Protein Activating Protease Taspase1

**DOI:** 10.1371/journal.pone.0151431

**Published:** 2016-03-14

**Authors:** Johannes van den Boom, Franziska Trusch, Lukas Hoppstock, Christine Beuck, Peter Bayer

**Affiliations:** 1 Department of Structural and Medicinal Biochemistry, Centre for Medical Biotechnology (ZMB), University of Duisburg-Essen, Essen, Germany; 2 Aberdeen Oomycetes Laboratory, Institute of Medical Sciences, University of Aberdeen, Aberdeen, United Kingdom; Russian Academy of Sciences, Institute for Biological Instrumentation, RUSSIAN FEDERATION

## Abstract

Type 2 asparaginases, a subfamily of N-terminal nucleophile (Ntn) hydrolases, are activated by limited proteolysis. This activation yields a heterodimer and a loop region at the C-terminus of the α-subunit is released. Since this region is unresolved in all type 2 asparaginase crystal structures but is close to the active site residues, we explored this loop region in six members of the type 2 asparaginase family using homology modeling. As the loop model for the childhood cancer-relevant protease Taspase1 differed from the other members, Taspase1 activation as well as the conformation and dynamics of the 56 amino acids loop were investigated by CD and NMR spectroscopy. We propose a helix-turn-helix motif, which can be exploited as novel anticancer target to inhibit Taspase1 proteolytic activity.

## Introduction

Proteolysis is a common regulatory process governing several essential pathways such as apoptosis [1] or blood clotting [2]. In contrast to protein digestion and degradation, proteolytic activation events occur in a site-specific manner and induce a conformational change that increases the catalytic activity significantly [3]. This type of regulation is common for proteases, but is also found among N-terminal nucleophile (Ntn) hydrolases involved in diverse cellular processes.

Ntn hydrolases are typically activated from an enzymatically inactive zymogen by hydrolysis of the peptide bond N-terminal of the active site residue. This intramolecular reaction occurs spontaneously and the new N-terminal amino acid (serine, threonine or cysteine) becomes the active site residue, located at the beginning of a β-strand [4,5]. Despite low sequence homology, diverse substrate specificity and functions, all Ntn hydrolases share a common αββα-asparaginase fold.

One subfamily of Ntn hydrolases is represented by type 2 asparaginase proteins, which comprise three different types of enzymes: (i) asparaginases convert l-asparagine to l-aspartate, (ii) glycosylasparaginases are involved in the degradation of glycoproteins, as they remove N-acetylglucosamine attached to asparagine, and (iii) threonine aspartase 1 (Taspase1) is a threonine protease responsible for the activation of the mixed lineage leukemia (MLL) protein involved in childhood leukemias [6], and regulates transcription factor IIa (TFIIa) [7].

Usually, inhibitory propeptides are completely removed during enzyme activation [8,9]. However, this is not the case for the above mentioned members of type 2 asparaginases [10–14], in which the activation releases a loop region at the C-terminus of the α-subunit. This loop comprises 10 to 56 amino acids and is not resolved in any type 2 asparaginase crystal structure due to a lack of electron density for this region, indicating high flexibility [10–14].

Essentially, the close proximity to the active site of this unresolved region and especially its crucial role for the activation of the cancer-related protease Taspase1 characterizes this region as a novel target for Taspase1 inhibition. The applicability as drug target is further underlined, since it comprises a nuclear localization signal (NLS), which was predicted *in silico* and confirmed *in vivo* [15]. Other type 2 asparaginase proteins, however, do not exhibit this NLS according to the sequence-based PredictNLS algorithm [16]. Indeed, these proteins were experimentally found in the periplasm (*E*. *coli* asparaginase and *F*. *meningosepticum* glycosylasparaginase [17]), the cytoplasm (human asparaginase; [18]) or lysosomes (human glycosylasparaginase; [19]).

As Taspase1 inhibitors have been reported to reduce breast cancer growth in mice [20], Taspase1 inhibitors have a promising potential in general cancer treatment. Here, we investigate the structure of six type 2 asparaginase loops at the C-terminus of the α-subunit *in silico* and present a spectroscopy supported model of this region in Taspase1.

## Material and Methods

### Secondary structure prediction

Secondary structure was predicted based on the protein sequence of human Taspase1 (RefSeq NP_060184.2) using Jpred [21], the Chou-Fasman algorithm (CFSSP; [22]), and the YASARA algorithm with default parameters.

### Homology modeling and molecular dynamics (MD) simulations

**Fig 1:**
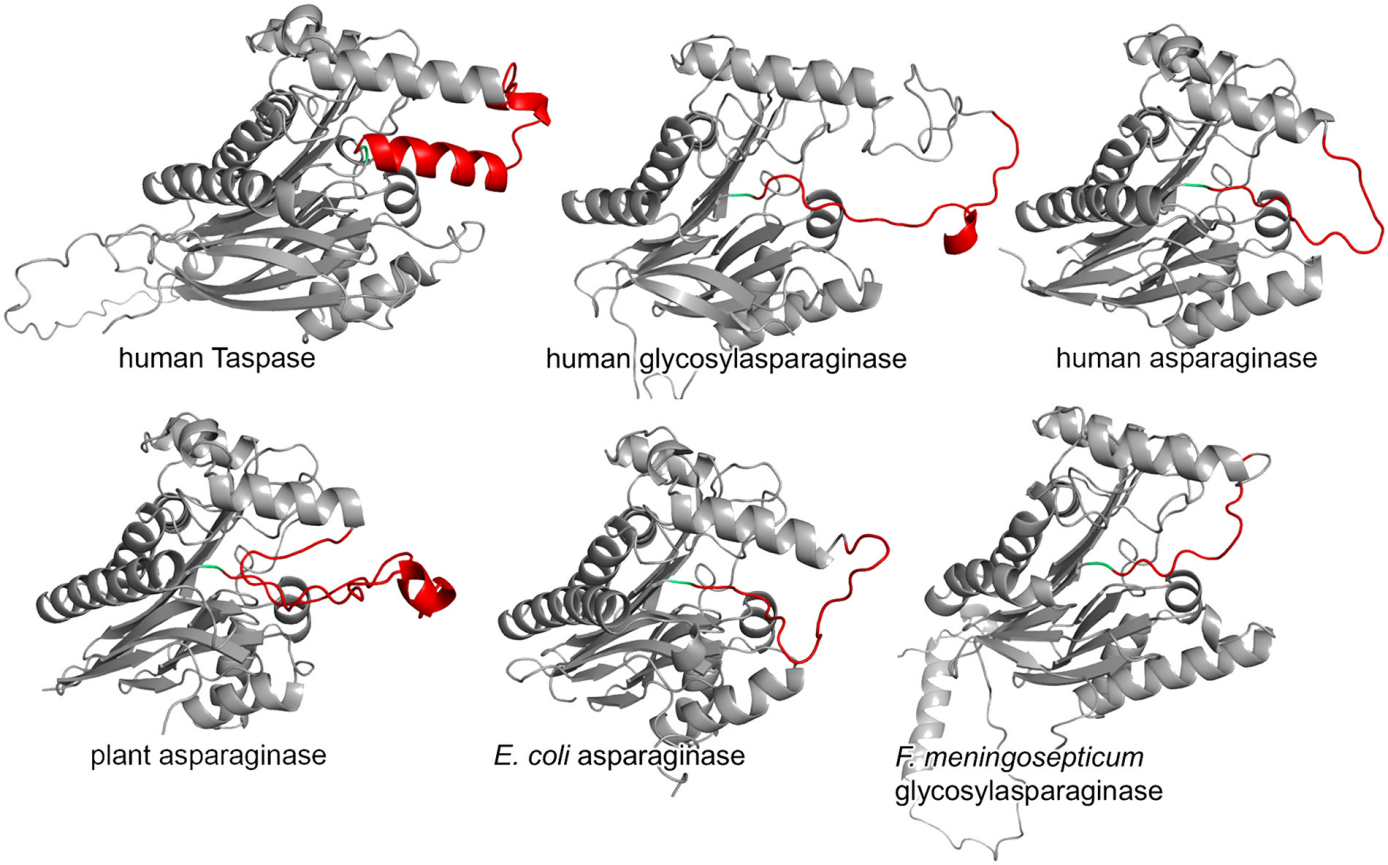
Type 2 asparaginase loop models. Modeled structures of the Taspase1 loop homologous regions in other type 2 asparaginases generated with YASARA. The loop of human Taspase1 (template PDB 2a8i; 25 modeled amino acids) is predicted as predominantly helical, while for human glycosylasparaginase (template PDB 1apy; 20 modeled aa), human asparaginase (template PDB 4pvs; 14 modeled aa), plant asparaginase (template PDB 2gez; 33 modeled aa), *E*. *coli* asparaginase (template PDB 2zal; 17 modeled aa), and *F*. *meningosepticum* glycosylasparaginase (template PDB 1ayy; 10 modeled aa) mainly random coil elements are predicted. The modeled loop regions are highlighted in red; the active site (corresponding to Thr234 in Taspase1) is highlighted in green.

Homology models including also flexible amino acids of the loop and the termini were generated with the YASARA Structure suite applying the parameters listed in S1 Table using the proenzyme crystal structures as templates. Unless otherwise described, the full amino acid sequence was used as query, therefore generating the proenzymes before proteolytic activation. The open state model of Taspase1 was obtained by modeling the two subunits with subsequent energy minimization of the resulting heterodimer. Three 10 ns all atoms molecular dynamics simulations of the Taspase1 loop fragment were performed applying a YAMBER3 [23], YASARA2 [24], or AMBER03 [25] force field with the YASARA Structure suite in a cubic simulation cell with 17.3 nm length and periodic boundaries. After energy minimization and simulated annealing (2 fs steps atom velocities scaled down to 90% every 20 fs; convergence with less than 0.05 kJ/mol per atom during 200 steps), MD simulations were started using default parameters and drift correction (1 fs time steps; 25°C; 0.8 nm Coulomb and van der Waals interactions cut off; particle-mesh Ewald calculations for long range Coulomb interactions; no angle or bond constraints). Open state loops of the regions missing in the active Taspase1 crystal structure PDB 2a8j and the respective regions in the homologous type 2 asparaginases were modelled using the YASARA algorithm for loop building. In short, the algorithm searches a non-redundant subset of the protein data bank for homologous sequences and extracts their conformations. These conformations were ranked for quality, fitted to the anchor points and the side chains were optimized. Ten models were generated per template to evaluate different possible conformations. Statistics for the secondary structure of the ten models are given in the main text and the top-ranked conformation was chosen for visualization in Fig 1.

### Cloning, protein expression and purification

His-tagged wild-type Taspase1 was cloned, expressed and purified as described before [26]. After expression in *E*. *coli* strain BL21(DE3)T1r in 4 l minimal medium (M9), supplemented with ^15^NH_4_Cl and/or ^13^C-glucose, for 6 h at 30°C, the soluble protein fraction after sonication and ultracentrifugation was loaded onto a HisTrap HP 5 ml Ni-NTA column (GE Healthcare, Munich, Germany) equilibrated with 50 mM NaH_2_PO_4_, pH 8.0, 450 mM NaCl, 10 mM imidazole. Fractions after imidazole elution containing Taspase1 were gel filtrated on a Superdex 200 HiLoad 16/600 column (GE Healthcare). Finally, fractions from the size exclusion chromatography containing Taspase1 were concentrated to 18 mg/ml.

The Taspase1 loop (Gly178-Asp233) was amplified by PCR (forward primer: 5'-CACACAGGGCCCGGAATACCGTCTTGCCCTCC; reverse primer: 5'-GGTTGGCTCGAGCTTAGTCCAAAGTGCCTGAGTCGTTC) and ApaI/XhoI-cloned into a modified pET41b vector containing an N-terminal GST tag and a PreScission protease cleavage site [27]. After expression in *E*. *coli* BL21(DE3)T1r at 25°C overnight, the soluble protein fraction after sonication and ultracentrifugation was loaded onto a 20 ml GSTrap column (GE Healthcare) equilibrated with 50 mM NaH_2_PO_4_, pH 7.0, 450 mM NaCl, 1 mM DTT, 1% Triton X-100. Protein containing fractions after glutathione elution were pooled and the GST-tag was cleaved off by incubation with PreScission protease (1 μg/mg fusion protein) overnight at 4°C. Separation of the GST-tag from the inhibitory peptide fragment was done by filtration through a membrane with 10 kDa cut off. The flow through containing the inhibitory peptide fragment was concentrated to 7 mg/ml.

### NMR spectroscopy

NMR spectra were recorded with a 700 MHz UltraShield NMR spectrometer (Bruker, Rheinstetten, Germany) equipped with a cryoprobe (Bruker Biospin). Samples contained 600 μM Taspase1 in 600 μl 50 mM NaH_2_PO_4_, pH 7.9, 300 mM NaCl, 1 mM DTT buffer supplemented with 2% D_2_O. Spectra for assignment of the full-length Taspase1 (^1^H-^15^N HSQC, HBHANH, HBHACONH, HNCA, HNCACB, ^1^H-^15^N TOSCY-HSQC as well as HCCH-TOCSY) were recorded at 30°C. For the ^13^C-^15^N-labelled Taspase1 loop (Gly178-Asp233; 150 μM in 50 mM KH_2_PO_4_, pH 6.5, with 10% D_2_O), ^1^H-^15^N HSQC, HNCACB, CBCACONH and ^1^H-^15^N TOSCY-HSQC spectra were taken at 25°C. All spectra were referenced using DSS for ^1^H and IUPAC for ^13^C and ^15^N [28]. Prior to processing a shifted sine-bell square window function for apodization was used. Processing was done using the Topspin 3.0 software (Bruker). Peak assignment was performed in CCPN and example sections of the 3D spectra used for chain tracing are shown in S1 Fig.

### CD spectroscopy

Far-UV CD spectra were recorded with a Jasco J-710 spectropolarimeter (Jasco, Gross-Umstadt) with 0.15 mg/ml protein in 50 mM NaH_2_PO_4_, pH 7.9 buffer at 21°C in 1 mm cuvettes. A buffer baseline was subtracted and units were converted to specific ellipticity. Secondary structure content was evaluated using the CDSSTR algorithm with the SDP48 reference data set.

## Results

### Asparaginase type 2 loop structure models

Homology models for the unresolved regions in six asparaginase type 2 proenzyme crystal structures (generated with YASARA) suggest that helices can establish in the respective regions (Fig 1). However, as these helices are typically short and occur only in a fraction of the loop models for asparaginases (53%) and glycosylasparaginases (45%), they are presumably transient. In contrast, the Taspase1 loop (Gly178-Asp233) is predicted to form helices in all models. Moreover, Taspase1 differs from other type 2 asparaginase family proteins in that its substrates are polypeptide bonds instead of the modification of single amino acids and that it does not cleave itself only but also acts as a protease *in trans* [6]. However, although Taspase1 holds a special position among type 2 asparaginases, they all share a very high degree of structural homology, especially with respect to the active site, and it is still unclear why only Taspase1 is an active protease. Hence, investigating the loop region next to the catalytic site might help to explain functional differences in the type 2 asparaginase family.

Interestingly, the amino acid sequence of the Taspase1 loop is highly conserved among Taspase1 proteins from 32 species (S2 Fig), but does not occur in other proteins, as BLAST searches retrieve only Taspase1 homologues, which indicates an essential and unique part of the protein. Hence, we focused our analyses on the Taspase1 loop and investigated its conformation.

**Fig 2:**
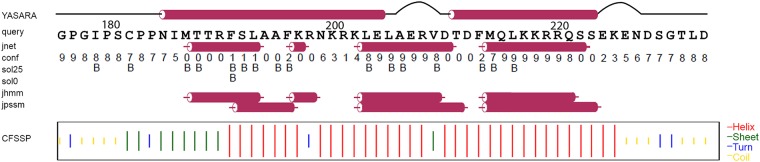
Secondary structure predictions of Taspase1 loop. Primary sequence based secondary structure predictions of the loop region Gly178-Asp233 of Taspase1, which is missing in the crystal structure of active Taspase1 (PDB 2a8j). The algorithms predict a stretch of helices between Asn185 and Ser223. For the YASARA model and the JNet prediction, cylinders represent helical areas, lines represent random coil and loops are represented as curves. Confidence (conf) values range from 0 (uncertain) to 9 (confident). Residues predicted as buried by sol25 and sol0 are labeled (B).

Sequence-based secondary structure predictions of the Taspase1 loop suggest a helical content of around 50% (Fig 2). In particular, both N- and C-terminus are predicted as random coil regions, while the central part (Asn185-Ser223) is mostly helical.

### The Taspase1 loop adopts a helix-turn-helix conformation

To test Taspase1 loop predictions *in vitro*, we exploited NMR spectroscopy to obtain structural data on this flexible region in the context of the full-length protein. Owing to the high molecular weight of Taspase1 forming a αββα-tetramer in solution (~90kDa) line broadening due to high correlation time and high number of hydrogen atoms should lead to a disappearance of most signals in a corresponding ^1^H-^15^N-HSQC spectrum. Only NH groups of very dynamic regions such as loops and termini are expected to generate measurable peaks.

**Fig 3:**
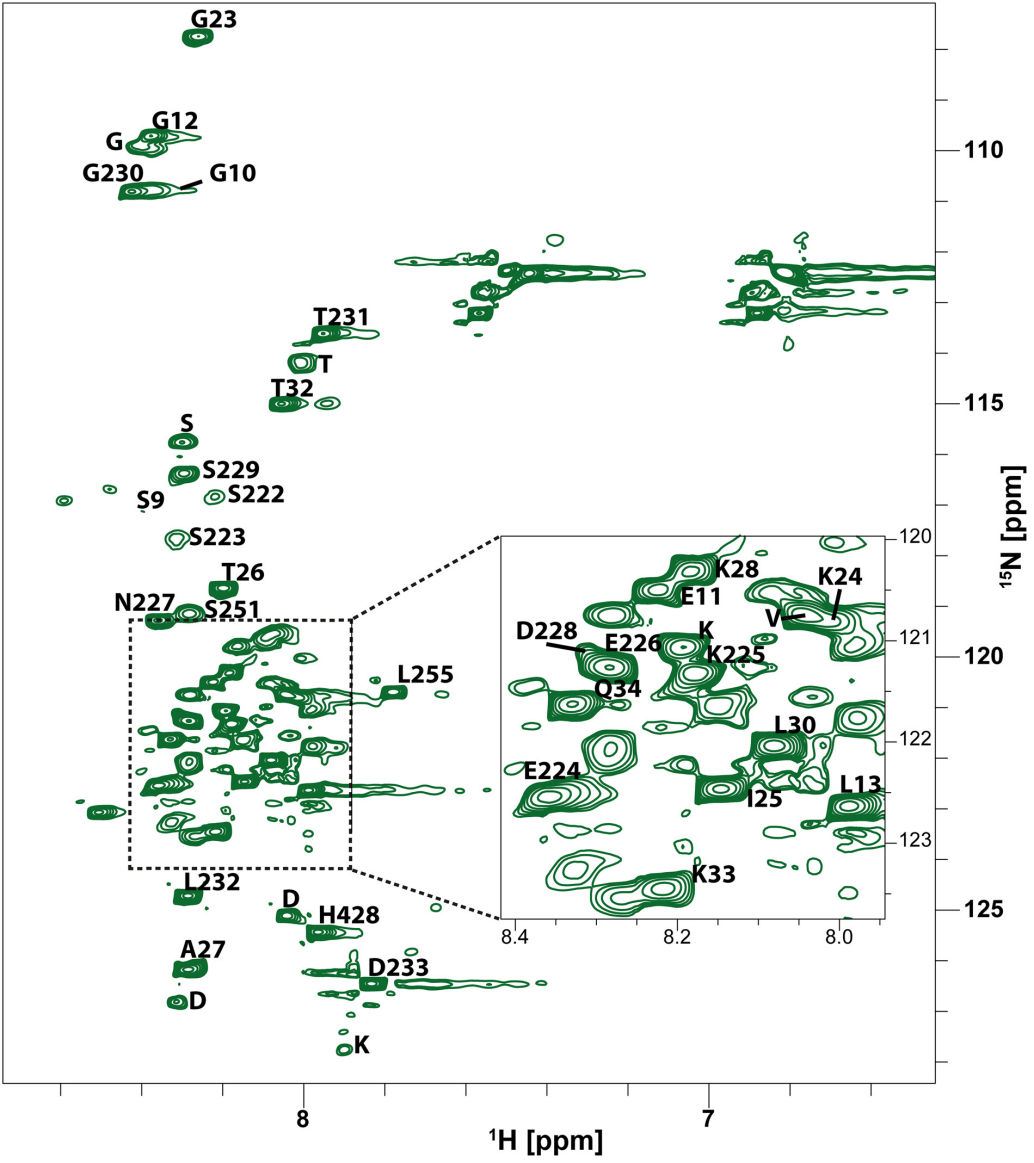
^1^H-^15^N HSQC of full-length Taspase1. ^1^H-^15^N HSQC spectrum of full-length Taspase1 after one week at 30°C. The crowded region in the center of the spectrum (dashed lines) is magnified. For amino acids labeled with letters without numbers, only the residue type could be determined unambiguously.

Hence, 56 peaks are visible in the ^1^H-^15^N HSQC spectrum of Taspase1, 32 of which were assigned unambiguously (Fig 3 and S2 Table). The low ^1^H signal dispersion of 0.8 ppm typical for intrinsically disordered proteins [29–31] indicates the absence of very rigid secondary structure elements in the observed regions.

Remarkably, the amino acids whose signals are observed are predominantly located either in the flexible N-terminus (15 residues) of Taspase1 or in the C-terminus (12 residues) of the α-subunit. Whereas ^1^H-^15^N signals for residues C-terminal of Arg220 could be assigned, no signals could be assigned to amino acids of the N-terminal part of the Taspase1 loop (Gly178 to Arg220). This indicates an interaction of this region to the protein core, probably due to the occurrence of a more rigid secondary structure element N-terminal of Glu221 with an increased rotational correlation time.

Furthermore, the increased flexibility of the Taspase1 loop upon cleavage is also reflected in the time dependence of NMR spectra: Right after purification, only the strongest signals from three amino acids (Glu226, Leu232, and Asp233) located in the loop are visible. This corresponds to the low amount of activated Taspase1 and concomitantly on average a less flexible loop. However, as activation proceeds, another nine signals from loop amino acids (Ser222-Lys225 and Asn227-Thr231) become visible over seven days, which is not due to simple protein degradation (S3 Fig).

**Fig 4:**
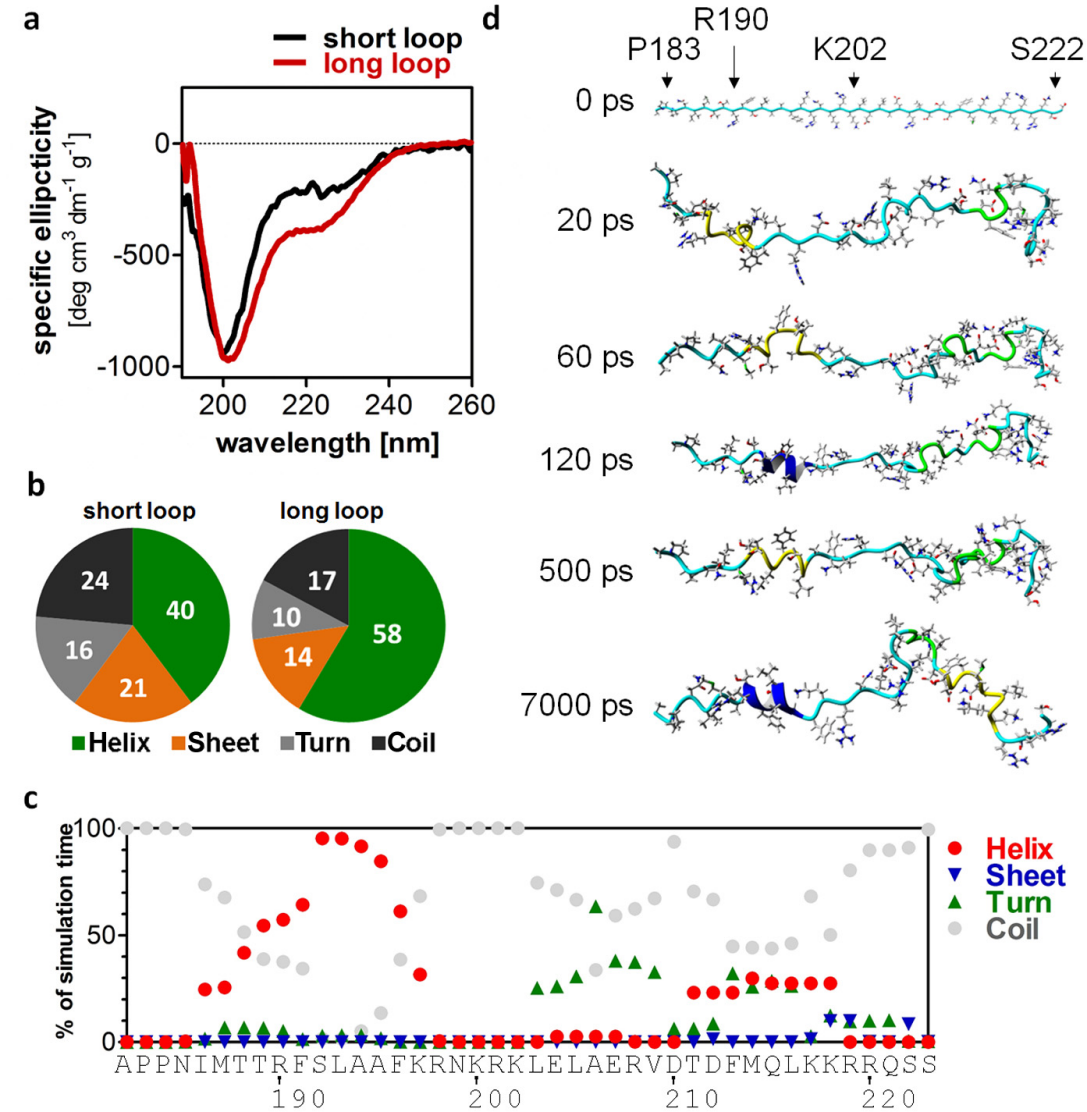
Spectroscopic analysis and MD simulation of the Taspase1 loop at the C-terminus of the α-subunit. (a) CD spectra of the long (G178-D233; red) and the short Taspase1 loop (P183-S222; black) show a helical structure for both peptides. (b) CD spectrum based secondary structure deconvolution using CDSSTR confirms a higher helical content (green) in the long loop compared to the short loop. Numbers are given in percent. (c) Three 10 ns molecular dynamics simulations were performed in YASARA starting with a linear loop peptide. For each amino acid, the time of the respective amino acid in helix, sheet, turn or random coil conformation is plotted. (d) Representative snapshots of the simulations indicate the formation of helices, especially around Arg190.

For further analyses, we expressed and purified a peptide covering all amino acids invisible in the crystal structure of activated Taspase1 (pdb 2a8j; Gly178-Asp233). Far-UV CD spectra of this peptide confirmed a significant helical content of 58%, which is in agreement with the secondary structure prediction (Fig 4a and 4b). Additionally, a shorter synthetic peptide covering the predicted helical center of the loop only (Pro183-Ser222) still retains these helical elements (Fig 4a and 4b). Molecular dynamics simulations with different force fields indicate that especially the second helix (around Leu216) forms only transiently in the short peptide (Fig 4c and 4d). Moreover, the simulations suggest that the stretch around Arg190 (first helix) is most prone to helix formation, while the terminal regions of the loop remained without any secondary structure elements. Importantly, the central part around Ala206 between the two helices formed a turn motif in all three simulations.

**Fig 5:**
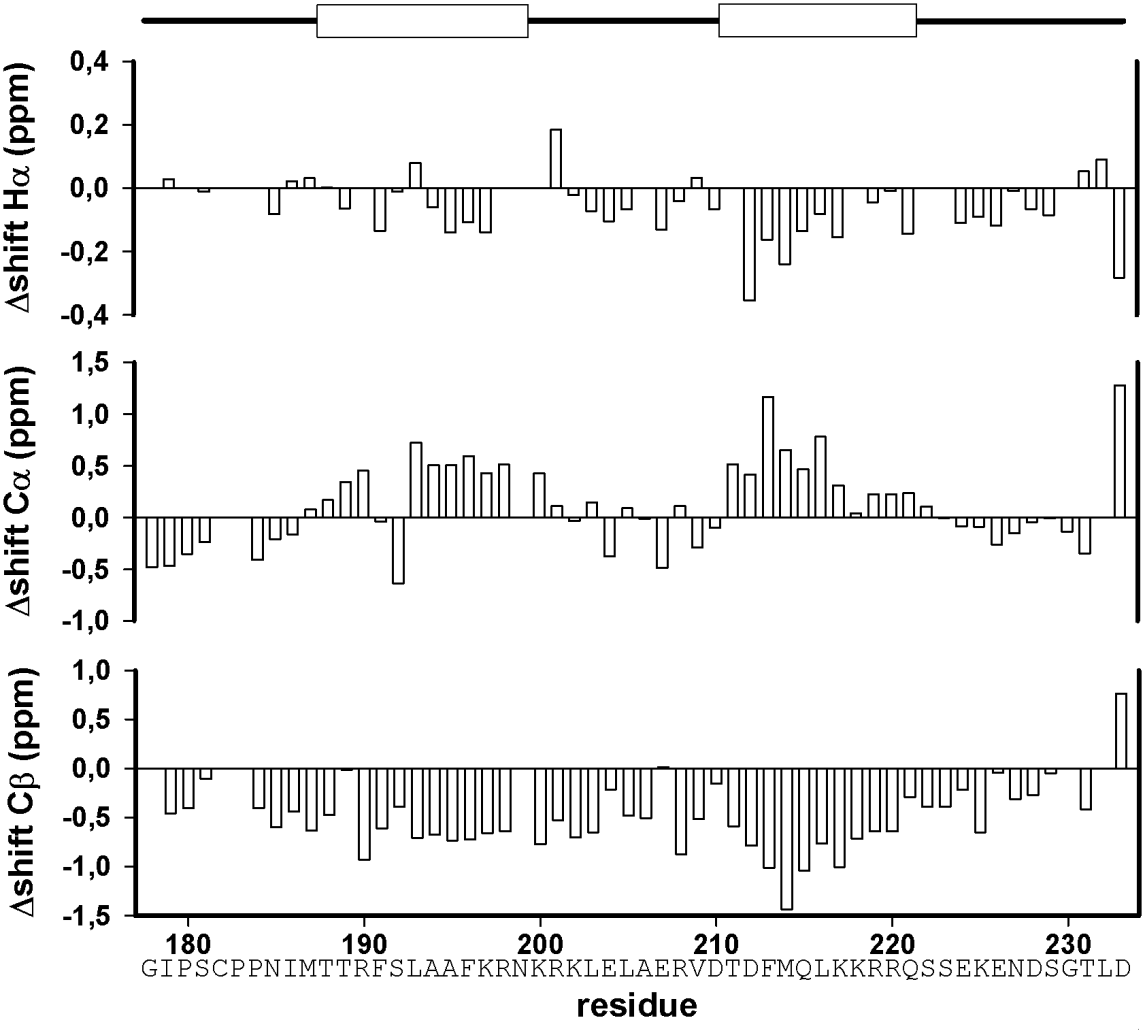
Chemical shift difference analysis of the long Taspase1 loop. Analysis of the chemical shift differences with respect to random coil values for H_α_, C_α_, and C_β_ shifts of the Taspase1 loop. Helices according to the secondary structure prediction are depicted on top. Note that stretches of negative H_α_ and C_β_ values, as well as positive C_α_ values indicate a helical conformation of the respective amino acids.

To verify these regions of secondary structure found in our simulations, we recorded NMR spectra of the isotope labeled long Taspase 1 loop (Gly178-Asp233). The lower pH value used for NMR (pH 6.5) compared to CD spectra (pH 7.9) represents a compromise between spectra quality (slower exchange rate of HN protons leads to better signal intensity) and helix stability (low pH destabilized the helices in CD spectra; data not shown). Since the transient character of the secondary structure elements precluded obtaining long range NOEs suitable for structure calculation, we investigated the appearance of secondary structure elements by the well-established approach according to Wishart [32]. The negative shift differences found in the H_α_- and C_β_-plots (Fig 5) as well as corresponding stretches of positive values in the C_α_ plot for Ile186-Asn199 and Thr211-Gln221 indicate transient helices occurring in the Taspase1 loop. Notably, the turn element (Lys200-Asp210) between both helices can clearly be identified by the alternating bars in the plots within this region.

**Fig 6:**
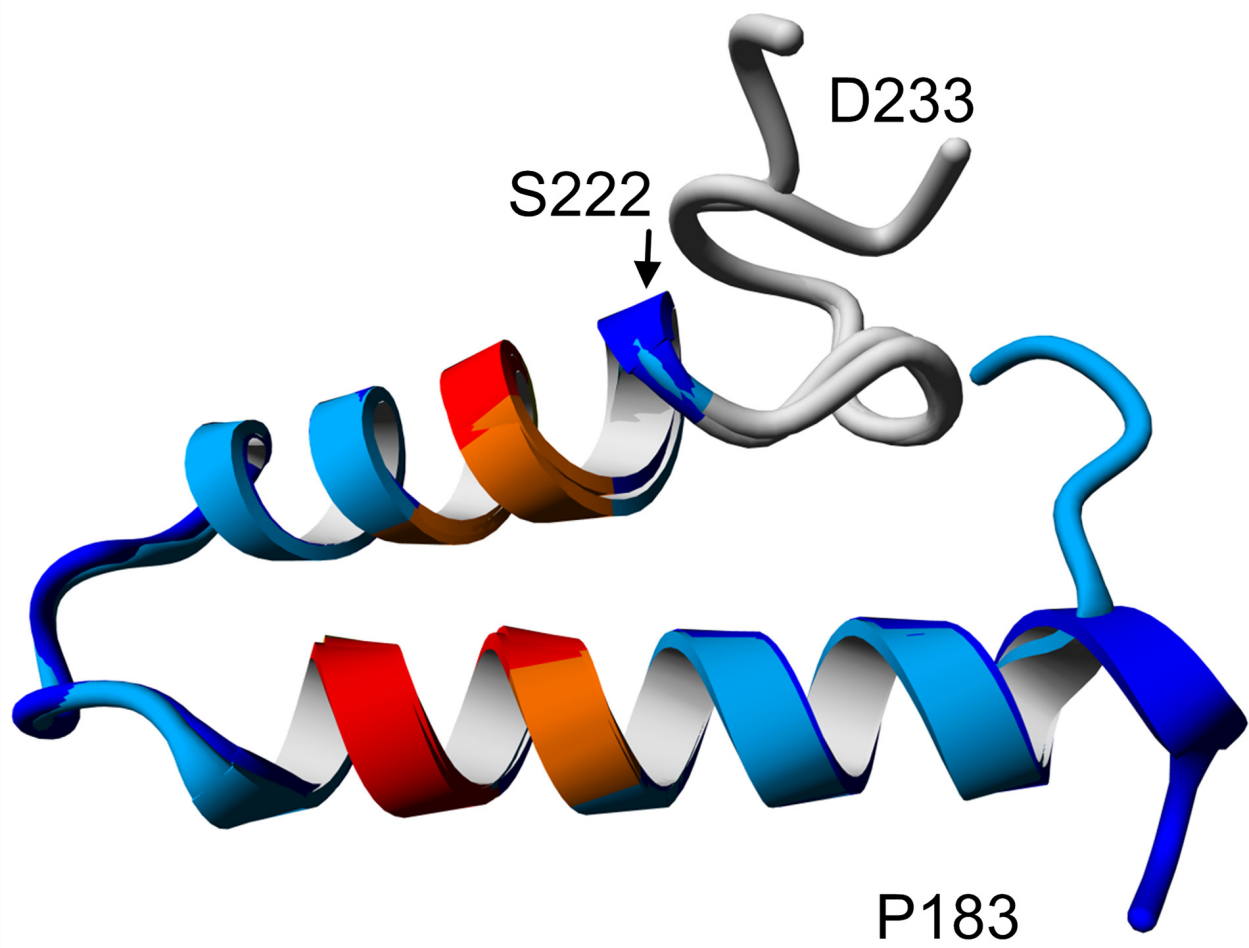
Proposed helix-turn-helix structure of Taspase1 loop. The helical character of the proposed structure is confirmed by CD spectroscopy and the detailed helix-turn-helix structure is supported by NMR spectroscopy data as well as secondary structure prediction. The model shows a helix-turn-helix motif. NLS amino acids are highlighted in orange, critical NLS residues are displayed in red (according to [15]). The overlay of two possible conformations indicates flexibility of the termini.

Finally, our data are in line with a helix-turn-helix model generated by homology modeling of the Taspase1 loop (Fig 6 and S1 file). This model matches the experimentally determined central turn region with two adjacent helices (Ile186-Leu203 and Asp210-Ser222), as well as a defined secondary structure element N-terminal of Ser222.

## Discussion

### Conformational changes of the Taspase1 loop upon activation

The autoproteolytic activation of type 2 asparaginase proteins causes major changes in a loop fragment at the α-subunit C-terminus and concomitantly greatly increases the flexibility in this region.

Here, we predicted a helical structure of this loop with a subsequent experimental *in vitro* confirmation. Essentially, our data support the model of an α-helical loop with a defined secondary structure that behaves independently of the Taspase1 core, suggesting a closed state for the zymogen and an open state for active Taspase1 (S4 Fig). This hypothesis is further supported by the absence of the respective NMR signals (Ser222, Lys225, Asn227, Ser229-Asp233) in a cleavage-deficient Asp233A/Thr234A mutant (S5 Fig), indicating a more rigid position of the very end of the α-subunit C-terminus in the mutant. However, in wild-type Taspase1, opening of the catalytic pocket is likely to be promoted by a repulsion of the positively charged loop from the also positively charged active site (in S6A and S6B Fig). As the two loops of the functional Taspase1 homodimer are located at opposite sides of the dimer (S6c Fig), the two opening events are unlikely promoted by an interaction of the two loops.

While homology modeling of the Taspase1 loop in the context of the full-length enzyme results in models with two adjacent helices (80% of all models), this is not the case for other type 2 asparaginases. Their loops appear to be less structured with a short single helix (22% of the models), interspersed non-adjacent helices (24%), or pure random coil (44%). Given the good agreement of the *in silico* with the experimental data, our methods could also be applied to the flexible parts of other type 2 asparaginases unresolved in the crystal structures. We expect comparable results by NMR spectroscopy which can aid to confirm predictions and to understand the structure and role of these loop regions also in other classes of enzymes.

### The Taspase1 loop represents a novel anti-cancer target

Taspase1 has been characterized as a disease-related protease [33,34] which is upregulated in a variety of tumors, including leukemia [35], breast and brain cancer [20]. Over the past years, Taspase1 has moved into the focus as an anticancer drug target, since inactivation has been shown to block tumor growth and initiation [20,36].

In our proposed helix-turn-helix model of the Taspase1 loop, the interaction of the two helices brings the bipartite nuclear localization signal (NLS) [15] in close vicinity and forms a continuous patch (red in Fig 6). Masking of an NLS to prevent catalytic activity was previously successfully demonstrated with an anti-NLS peptide binding to the HIV-1 integrase NLS, thus preventing its interaction with importin α and consequently HIV reproduction [37,38]. Hence, impeding nuclear uptake of Taspase1 is a new approach to inhibit Taspase1 activity, particularly because the maturation event requires a nuclear localization of Taspase1.Therefore, a decreased nuclear import does not only spatially separate Taspase1 from its nuclear target proteins, but also prevents Taspase1 activation, as cytosolic Taspase1 does not undergo proteolytic maturation and remains catalytically inactive [15].

Interestingly, Taspase1 is insensitive towards general protease inhibitors, but inhibition by a natural bisarsenic compound with broad bioactivity [20] as well as weak inhibition by substrate analog vinyl sulfone peptides have been shown recently [39]. With regard to the high need of potent Taspase1 inhibitors, the loop might represent a novel target to combat Taspase1-dependent tumors, such as mixed lineage leukemia. As the Taspase1 loop is in proximity of the active site, inhibitors binding the loop region could act as a clamp to keep the loop in a state similar to the closed proenzyme, which has already been successfully exploited for caspases [40]. Alternatively, the autocatalytic activation event could be prevented by binding of inhibitors, as this can prevent Taspase1 from adopting the conformation required for the nucleophilic attack initiating self-cleavage.

These approaches render the Taspase1 loop a promising novel anticancer target, and we expect that our structural model for this region opens up new approaches for inhibitor design.

## Supporting Information

S1 Fig Example sections of 3D NMR spectra used for sequential assignment. Assignment of ^1^H-^15^N HSQC peaks was accomplished with the help of 3D spectra. As an example, the sequential connections (dashed lines) between Hα and Hβ atoms (top panel), as well as Cα and Cβ atoms are shown for the assignment of the stretch between Asn227 and Asp233, located at the tip of the Taspase1 loop. In the upper panel, positive and negative HBHANH peaks are displayed in green and red, respectively; HBHACONH signals are displayed in blue. In the lower panel, positive and negative HNCACB peaks are shown in green and red, respectively; CBCACONH signals are displayed in blue.
(PDF)

S2 Fig Conservation of the Taspase1 loop sequence. The sequence logo is based on a MUSCLE multiple sequence alignment of the loop regions of Taspase1 homologues from 32 species reveals high conservation. Blue: positively charged amino acids; green: neutral amino acids; black: hydrophobic amino acids; red: negatively charged amino acids.
(PDF)

S3 Fig Time dependent appearance of Taspase1 signals in NMR spectra. **(a)** ^1^H-^15^N HSQC spectra of Taspase1 were recorded right after purification (black) and after one week incubation at 30°C (red). Peaks of the spectrum right after purification are labeled. **(b)** SDS-PAGE analysis of Taspase1 samples after incubation at 37°C shows autocatalytic processing of full-length (fl) Taspase1 to α and β subunits. Note that no further degradation is visible.
(PDF)

S4 Fig Proposed open and closed states of Taspase1 loop. **(a)** Missing amino acids of the loop modeled into the crystal structure of the proenzyme (PDB 2a8i). In the proenzyme, the Taspase1 loop (red and orange) is covalently attached to the active site (green). The loop amino acids that are structured in the crystal structure of the proenzyme, but not in the processed protein are colored in orange. Newly modeled amino acids are displayed in red. **(b)** After autocatalytic activation, the loop (red) can leave the active site (green). The model is based on the structure of active Taspase1 (PDB 2a8j).
(PDF)

S5 Fig Comparison of wild-type and mutant Taspase1. ^1^H-^15^N HSQC spectra of wild-type Taspase1 (red) and inactive Taspase1 (blue) after 7 days incubation at 30°C. All backbone peaks of the inactive mutant can also be found in the spectrum of the wild-type protein. The 10 peaks visible only in the wild-type protein are labeled. 9 of these peaks are located in the loop which is released by autocatalytic activation. A labeling of all assigned peaks can be found in Fig 3.
(PDF)

S6 Fig Electrostatic potentials of Taspase1 and location of the Taspase1 loop. **(a)** Electrostatic potentials were calculated using the Adaptive Poisson-Boltzmann Solver (APBS) applying the YAMBER2 force field. Positive, neutral, and negative charges are displayed in blue, gray, and red, respectively. The surface charge of the Taspase1 alpha-subunit C-terminus is visualized for our proposed loop model and reveals a positive charge. **(b)** The surface charge of the Taspase1 core is visualized for the crystal structure of one active Taspase1 heterodimer (PDB 2a8j). The active site (dashed line) is also positively charged. **(c)** Location of the loop in the functional homodimer of Taspase1. Our proposed loop structure was modeled on both monomers of the Taspase1 proenzyme (PDB 2a8i). Monomer 1 is depicted in orange with its loop in green, monomer 2 in red with its loop in blue.
(PDF)

S1 File PDB coordinates of Fig 6. (PDB)

S1 Table Parameters for homology modeling in YASARA. (PDF)

S2 Table NMR shift values of Taspase1 full-length, given in ppm. (PDF)

S3 Table NMR shift values of the Taspase1 loop, given in ppm. (PDF)
