# Supplementary material for: Structural Characterization of the Loop at the Alpha-Subunit C-Terminus of the Mixed Lineage Leukemia Protein Activating Protease Taspase1
Source: PLoS One. 2016 Mar 14;11(3):e0151431. doi: 10.1371/journal.pone.0151431 (PMC4790943; doi:10.1371/journal.pone.0151431)
Supplement: S1 Fig — Assignment of 1H-15N HSQC peaks was accomplished with the help of 3D spectra. As an example, the sequential connections (dashed lines) between Hα and Hβ atoms (top panel), as well as Cα and Cβ atoms are shown for the assignment of the stretch between Asn227 and Asp233, located at the tip of the Taspase1 loop. In the upper panel, positive and negative HBHANH peaks are displayed in green and red, respectively; HBHACONH signals are displayed in blue. In the lower panel, positive and negative HNCACB peaks are shown in green and red, respectively; CBCACONH signals are displayed in blue. (PDF) [file pone.0151431.s001.pdf]

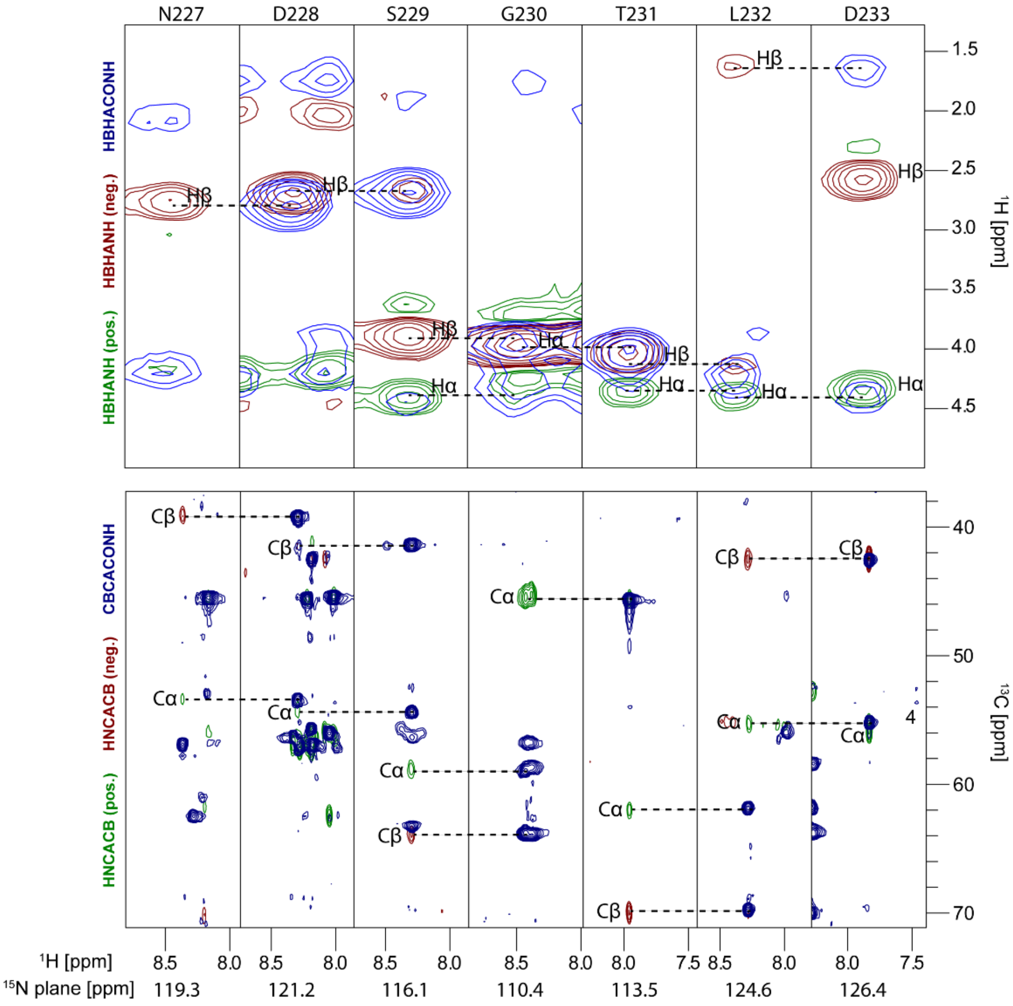

S1 Fig. Example sections of 3D NMR spectra used for sequential assignment.

Assignment of ^{1}H-^{15}N HSQC peaks was accomplished with the help of 3D spectra. As an example, the sequential connections (dashed lines) between Hα and Hβ atoms (top panel), as well as Cα and Cβ atoms are shown for the assignment of the stretch between Asn227 and Asp233, located at the tip of the Taspase1 loop. In the upper panel, positive and negative HBHANH peaks are displayed in green and red, respectively; HBHACONH signals are displayed in blue. In the lower panel, positive and negative HNCACB peaks are shown in green and red, respectively; CBCACONH signals are displayed in blue.
