# Supplementary material for: Structural Characterization of the Loop at the Alpha-Subunit C-Terminus of the Mixed Lineage Leukemia Protein Activating Protease Taspase1
Source: PLoS One. 2016 Mar 14;11(3):e0151431. doi: 10.1371/journal.pone.0151431 (PMC4790943; doi:10.1371/journal.pone.0151431)
Supplement: S2 Fig — The sequence logo is based on a MUSCLE multiple sequence alignment of the loop regions of Taspase1 homologues from 32 species reveals high conservation. Blue: positively charged amino acids; green: neutral amino acids; black: hydrophobic amino acids; red: negatively charged amino acids. (PDF) [file pone.0151431.s002.pdf]

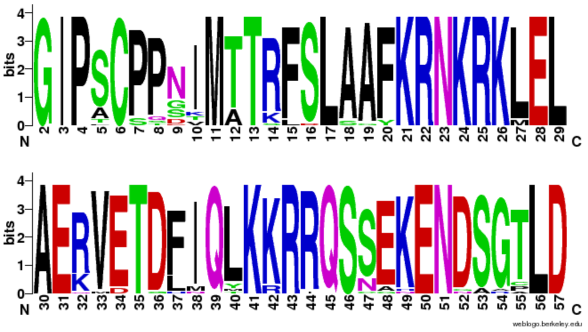

S2 Fig. Conservation of the Taspase1 loop sequence.

The sequence logo is based on a MUSCLE multiple sequence alignment of the loop regions of Taspase1 homologues from 32 species reveals high conservation. Blue: positively charged amino acids; green: neutral amino acids; black: hydrophobic amino acids; red: negatively charged amino acids.
