# Supplementary material for: Structural Characterization of the Loop at the Alpha-Subunit C-Terminus of the Mixed Lineage Leukemia Protein Activating Protease Taspase1
Source: PLoS One. 2016 Mar 14;11(3):e0151431. doi: 10.1371/journal.pone.0151431 (PMC4790943; doi:10.1371/journal.pone.0151431)
Supplement: S3 Fig — (a) 1H-15N HSQC spectra of Taspase1 were recorded right after purification (black) and after one week incubation at 30°C (red). Peaks of the spectrum right after purification are labeled. (b) SDS-PAGE analysis of Taspase1 samples after incubation at 37°C shows autocatalytic processing of full-length (fl) Taspase1 to α and β subunits. Note that no further degradation is visible. (PDF) [file pone.0151431.s003.pdf]

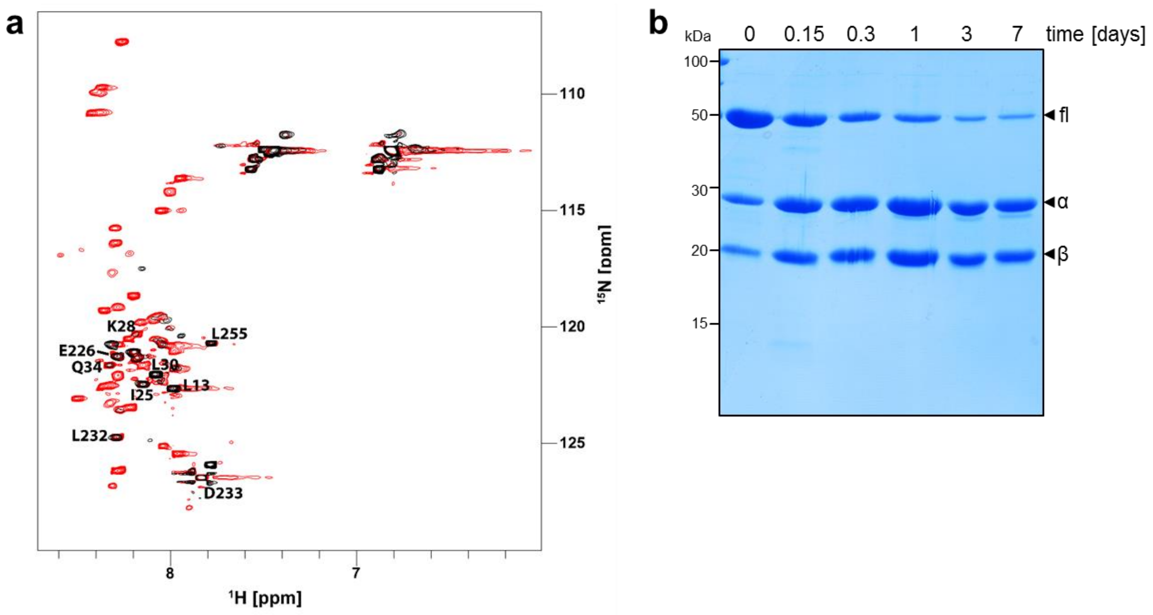


**S3 Fig. Time dependent appearance of Taspase1 signals in NMR spectra.**

**(a)** $^{1}$H-$^{15}$N HSQC spectra of Taspase1 were recorded right after purification (black) and after one week incubation at 30 °C (red). Peaks of the spectrum right after purification are labeled. **(b)** SDS-PAGE analysis of Taspase1 samples after incubation at 37 °C shows autocatalytic processing of full-length (fl) Taspase1 to α and β subunits. Note that no further degradation is visible.
