# Supplementary material for: Structural Characterization of the Loop at the Alpha-Subunit C-Terminus of the Mixed Lineage Leukemia Protein Activating Protease Taspase1
Source: PLoS One. 2016 Mar 14;11(3):e0151431. doi: 10.1371/journal.pone.0151431 (PMC4790943; doi:10.1371/journal.pone.0151431)
Supplement: S4 Fig — (a) Missing amino acids of the loop modeled into the crystal structure of the proenzyme (PDB 2a8i). In the proenzyme, the Taspase1 loop (red and orange) is covalently attached to the active site (green). The loop amino acids that are structured in the crystal structure of the proenzyme, but not in the processed protein are colored in orange. Newly modeled amino acids are displayed in red. (b) After autocatalytic activation, the loop (red) can leave the active site (green). The model is based on the structure of active Taspase1 (PDB 2a8j). (PDF) [file pone.0151431.s004.pdf]

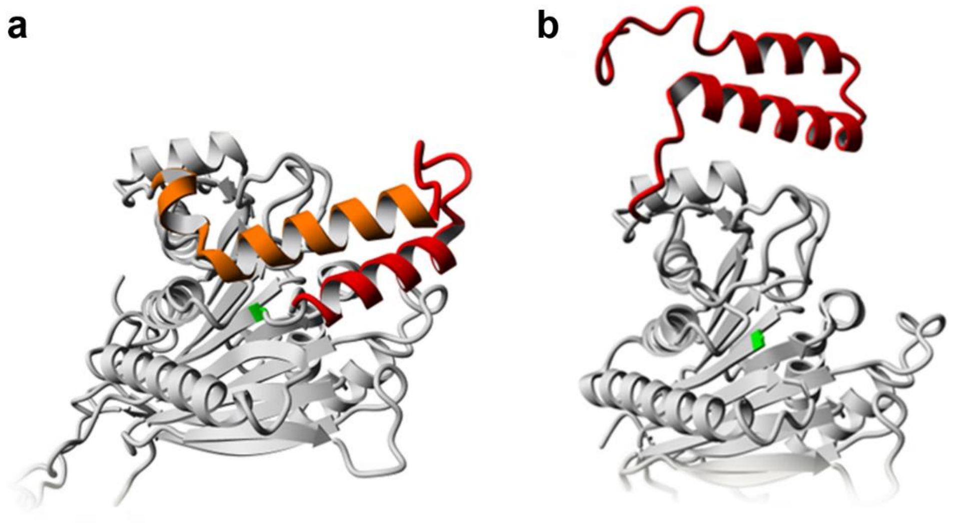

S4 Fig. Proposed open and closed states of Taspase1 loop.

(a) Missing amino acids of the loop modeled into the crystal structure of the proenzyme (PDB 2a8i). In the proenzyme, the Taspase1 loop (red and orange) is covalently attached to the active site (green). The loop amino acids that are structured in the crystal structure of the proenzyme, but not in the processed protein are colored in orange. Newly modeled amino acids are displayed in red. **(b)** After autocatalytic activation, the loop (red) can leave the active site (green). The model is based on the structure of active Taspase1 (PDB 2a8j).
