# Supplementary material for: Structural Characterization of the Loop at the Alpha-Subunit C-Terminus of the Mixed Lineage Leukemia Protein Activating Protease Taspase1
Source: PLoS One. 2016 Mar 14;11(3):e0151431. doi: 10.1371/journal.pone.0151431 (PMC4790943; doi:10.1371/journal.pone.0151431)
Supplement: S5 Fig — 1H-15N HSQC spectra of wild-type Taspase1 (red) and inactive Taspase1 (blue) after 7 days incubation at 30°C. All backbone peaks of the inactive mutant can also be found in the spectrum of the wild-type protein. The 10 peaks visible only in the wild-type protein are labeled. 9 of these peaks are located in the loop which is released by autocatalytic activation. A labeling of all assigned peaks can be found in Fig 3. (PDF) [file pone.0151431.s005.pdf]

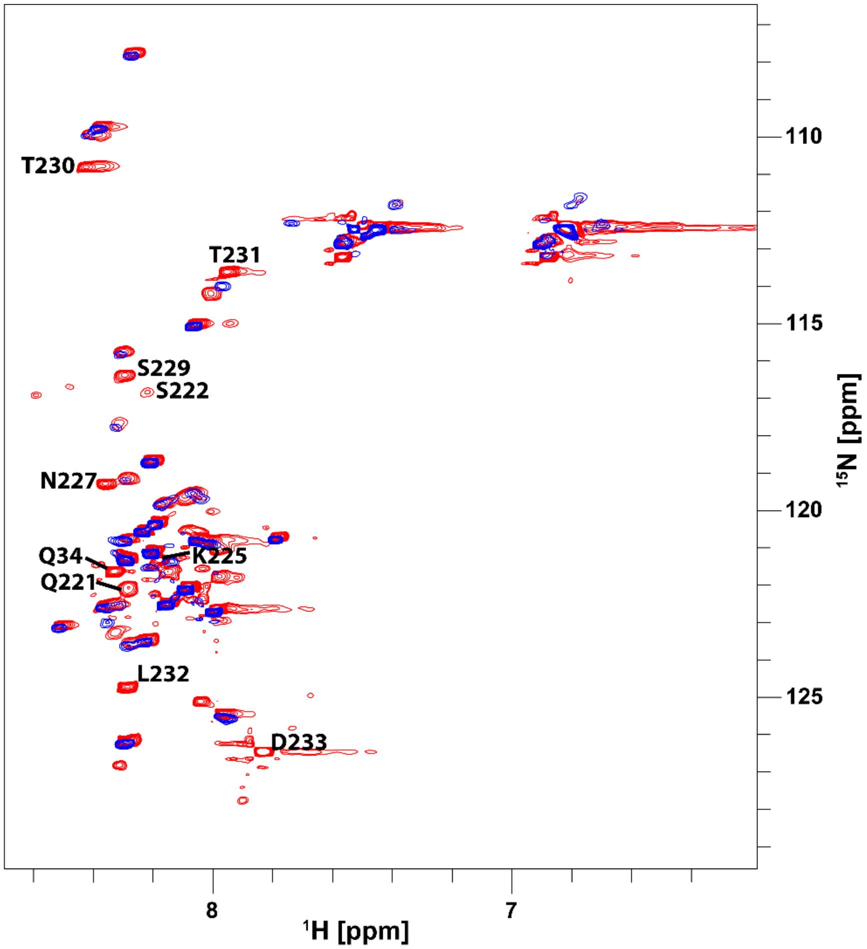

S5 Fig. Comparison of wild-type and mutant Taspase1.

^{1}H-^{15}N HSQC spectra of wild-type Taspase1 (red) and inactive Taspase1 (blue) after 7 days incubation at 30 °C. All backbone peaks of the inactive mutant can also be found in the spectrum of the wild-type protein. The 10 peaks visible only in the wild-type protein are labeled. 9 of these peaks are located in the loop which is released by autocatalytic activation. A labeling of all assigned peaks can be found in Fig 3.
