# Supplementary material for: Structural Characterization of the Loop at the Alpha-Subunit C-Terminus of the Mixed Lineage Leukemia Protein Activating Protease Taspase1
Source: PLoS One. 2016 Mar 14;11(3):e0151431. doi: 10.1371/journal.pone.0151431 (PMC4790943; doi:10.1371/journal.pone.0151431)
Supplement: S6 Fig — (a) Electrostatic potentials were calculated using the Adaptive Poisson-Boltzmann Solver (APBS) applying the YAMBER2 force field. Positive, neutral, and negative charges are displayed in blue, gray, and red, respectively. The surface charge of the Taspase1 alpha-subunit C-terminus is visualized for our proposed loop model and reveals a positive charge. (b) The surface charge of the Taspase1 core is visualized for the crystal structure of one active Taspase1 heterodimer (PDB 2a8j). The active site (dashed line) is also positively charged. (c) Location of the loop in the functional homodimer of Taspase1. Our proposed loop structure was modeled on both monomers of the Taspase1 proenzyme (PDB 2a8i). Monomer 1 is depicted in orange with its loop in green, monomer 2 in red with its loop in blue. (PDF) [file pone.0151431.s006.pdf]

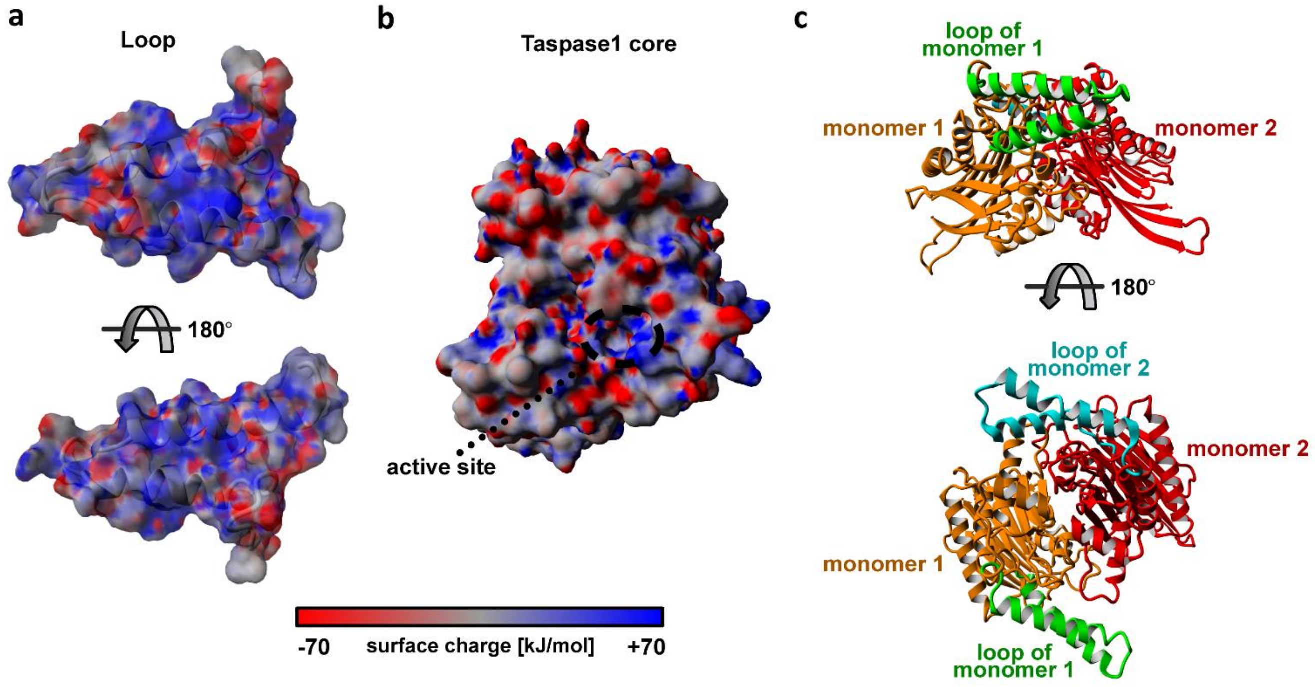

S6 Fig. Electrostatic potentials of Taspase1 and location of the Taspase1 loop.

(a) Electrostatic potentials were calculated using the Adaptive Poisson-Boltzmann Solver (APBS) applying the YAMBER2 force field. Positive, neutral, and negative charges are displayed in blue, gray, and red, respectively. The surface charge of the Taspase1 alpha-subunit C-terminus is visualized for our proposed loop model and reveals a positive charge. **(b)** The surface charge of the Taspase1 core is visualized for the crystal structure of one active Taspase1 heterodimer (PDB 2a8j). The active site (dashed line) is also positively charged. **(c)** Location of the loop in the functional homodimer of Taspase1. Our proposed loop structure was modeled on both monomers of the Taspase1 proenzyme (PDB 2a8i). Monomer 1 is depicted in orange with its loop in green, monomer 2 in red with its loop in blue.
