# Supplementary material for: Structural Characterization of the Loop at the Alpha-Subunit C-Terminus of the Mixed Lineage Leukemia Protein Activating Protease Taspase1
Source: PLoS One. 2016 Mar 14;11(3):e0151431. doi: 10.1371/journal.pone.0151431 (PMC4790943; doi:10.1371/journal.pone.0151431)
Supplement: S1 Table — (PDF) [file pone.0151431.s008.pdf]

S1 Table. Parameters for homology modeling in YASARA.

Parameter	Value
**PsiBLASTs**	**1**
**EValue Max**	**0.5**
**Templates Total**	**5**
**Templates SameSeq**	**1**
**OligoState**	**4**
**Alignments**	**5**
**LoopSamples**	**50**
**TermExtensions**	**90**
**Speed**	**Slow**
