# Supplementary material for: Structural Characterization of the Loop at the Alpha-Subunit C-Terminus of the Mixed Lineage Leukemia Protein Activating Protease Taspase1
Source: PLoS One. 2016 Mar 14;11(3):e0151431. doi: 10.1371/journal.pone.0151431 (PMC4790943; doi:10.1371/journal.pone.0151431)
Supplement: S2 Table — (PDF) [file pone.0151431.s009.pdf]

**S2 Table. NMR shift values of Taspase1 full-length, given in ppm.**

| aa | Res | HN | N | Cα | Cβ | Hα | Hβ | Hγ | Hδ | Hε |
|---|---|---|---|---|---|---|---|---|---|---|
| 9 | Ser | 8.35 | 117.1 | 58.62 | 64.11 | 4.47 | 3.86 | - | - | - |
| 10 | Gly | 8.35 | 110.7 | 45.40 | - | 3.92 | - | - | - | - |
| 11 | Glu | 8.19 | 120.5 | 56.83 | 30.26 | 4.23 | 1.96 | 2.16 | - | - |
| 12 | Gly | 8.35 | 109.8 | 45.25 | - | 3.89 | - | - | - | - |
| 13 | Leu | 7.96 | 122.7 | 53.19 | 41.74 | 4.57 | 1.56 | 1.50 | 0.86 | - |
| 23 | Gly | 8.23 | 107.7 | 45.40 | - | 3.88 | - | - | - | - |
| 24 | Lys | 7.99 | 120.8 | 56.34 | 33.11 | 4.27 | 1.69 | 1.35 | - | 3.00 |
| 25 | Ile | 8.12 | 122.5 | 61.29 | 38.78 | 4.17 | 1.78 | - | - | - |
| 26 | Thr | 8.17 | 118.7 | 61.91 | 70.20 | 4.29 | - | - | 1.13 | - |
| 27 | Ala | 8.25 | 126.2 | 53.01 | 19.12 | 4.20 | 1.33 | - | - | - |
| 28 | Lys | 8.15 | 120.3 | - | - | 4.18 | 1.68 | - | - | - |
| 32 | Thr | 8.03 | 115.0 | 62.28 | 69.86 | 4.23 | - | 1.06 | - | - |
| 33 | Lys | 8.19 | 123.4 | 56.65 | 32.61 | 4.23 | 1.71 | - | - | - |
| 34 | Gln | 8.30 | 121.7 | 56.89 | 30.18 | 4.18 | 1.94 | 2.17 | - | - |
| 222 | Ser | 8.18 | 116.9 | 58.56 | 63.92 | - | 3.78 | - | - | - |
| 223 | Ser | 8.29 | 117.7 | 58.80 | 64.02 | - | 3.87 | - | - | - |
| 224 | Glu | 8.34 | 122.6 | 56.85 | 30.10 | 4.22 | 1.75 | - | - | - |
| 225 | Lys | 8.15 | 121.3 | 56.66 | 33.01 | 4.23 | 1.71 | - | - | - |
| 226 | Glu | 8.25 | 121.3 | 57.23 | 29.88 | 4.17 | 1.99 | 2.19 | - | - |
| 227 | Asn | 8.33 | 119.3 | 53.43 | 39.18 | 4.13 | 2.74 | - | - | - |
| 228 | Asp | 8.27 | 121.2 | 54.42 | 41.52 | - | 2.64 | - | - | - |
| 229 | Ser | 8.26 | 116.3 | 58.94 | 64.02 | 4.40 | 3.85 | - | - | - |
| 230 | Gly | 8.40 | 110.7 | 45.57 | - | 3.93 | - | - | - | - |
| 231 | Thr | 7.93 | 113.6 | 62.00 | 70.07 | 4.29 | 4.00 | 1.14 | - | - |
| 232 | Leu | 8.25 | 124.8 | 55.27 | 42.44 | 4.35 | 1.58 | 1.6 | - | - |
| 233 | Asp | 7.80 | 126.5 | 55.87 | 42.55 | 4.30 | - | - | - | - |
| 251 | Ser | 8.25 | 119.1 | 58.55 | 63.88 | 4.36 | 3.81 | - | - | - |
| 428 | His | 7.98 | 125.4 | - | - | - | 3.06 | - | - | - |
