# Supplementary material for: Structural Characterization of the Loop at the Alpha-Subunit C-Terminus of the Mixed Lineage Leukemia Protein Activating Protease Taspase1
Source: PLoS One. 2016 Mar 14;11(3):e0151431. doi: 10.1371/journal.pone.0151431 (PMC4790943; doi:10.1371/journal.pone.0151431)
Supplement: S3 Table — (PDF) [file pone.0151431.s010.pdf]

S3 Table. NMR shift values of the Taspase1 loop, given in ppm.

aa	Res	HN	N	Cα	Cβ	Hα
178	Gly	8.55	109.59	44.62	-	3.99/3.75
179	Ile	8.08	121.76	58.24	34.24	4.5
180	Pro	-	-	62.95	31.69	-
181	Ser	8.38	115.85	58.06	63.69	4.46
182	Cys	8.48	121.62	52.84	40.08	4.20
183	Pro	-	-	-	-	-
184	Pro	-	-	62.89	31.69	-
185	Asn	8.48	118.19	52.89	38.30	4.66
186	Ile	8.07	120.83	60.94	38.36	4.19
187	Met	8.42	123.40	55.48	32.27	4.51
188	Thr	8.13	114.82	61.97	69.33	4.35
189	Thr	8.11	116.01	62.14	69.78	4.29
190	Arg	-	-	56.46	29.97	-
191	Phe	8.16	120.58	57.66	38.99	4.49
192	Ser	8.12	117.26	57.16	63.41	4.46
193	Leu	8.34	124.86	55.82	41.69	4.42
194	Ala	8.17	123.11	53.01	18.42	4.26
195	Ala	8.01	121.53	53.01	18.37	4.18
196	Phe	7.99	118.70	58.29	38.88	4.51
197	Lys	8.11	121.52	56.63	32.44	4.18
198	Arg	8.17	121.10	56.51	30.26	-
199	Asn	-	-	-	-	-
200	Lys	-	-	56.63	32.33	-
201	Arg	8.23	121.27	56.11	30.37	4.53
202	Lys	8.22	121.98	56.17	32.39	4.30
203	Leu	8.20	122.99	55.25	41.75	4.27
204	Glu	8.39	121.58	56.23	29.68	4.25
205	Leu	8.17	122.33	55.19	41.92	4.27
206	Ala	8.16	123.64	52.49	18.59	-
207	Glu	8.21	119.30	56.11	29.91	4.22
208	Arg	8.40	122.29	56.11	30.03	4.30
209	Val	8.23	120.83	61.91	32.38	4.15
210	Asp	8.46	123.79	54.10	40.94	4.57
211	Thr	8.09	114.10	62.32	69.21	-
212	Asp	8.38	122.47	54.62	40.31	4.29
213	Phe	8.15	120.71	58.87	38.59	4.46
214	Met	8.16	119.54	56.05	31.46	4.24
215	Gln	8.12	119.37	56.17	28.36	4.21
216	Leu	8.05	121.66	55.88	41.63	4.26
217	Lys	8.13	120.59	56.51	32.10	4.17
218	Lys	-	-	56.24	32.38	-
219	Arg	8.25	121.84	56.23	30.26	4.30
220	Arg	8.23	121.81	56.23	30.26	4.33
221	Gln	8.51	121.71	55.94	29.11	4.20
222	Ser	8.45	117.04	58.41	63.41	-
223	Ser	8.39	117.31	58.29	63.41	-
224	Glu	8.44	122.43	56.51	29.68	4.24
225	Lys	8.25	121.13	56.11	32.45	4.25
226	Glu	8.40	121.60	56.34	29.86	4.23
227	Asn	8.45	119.13	52.95	38.59	4.73
228	Asp	8.36	121.10	54.16	40.83	4.57
229	Ser	8.37	116.28	58.29	63.75	4.39
230	Gly	8.49	110.73	44.97	-	3.85
231	Thr	8.03	113.66	61.45	69.38	4.40
232	Leu	8.37	124.79	54.67	42.04	4.43
233	Asp	7.89	126.37	55.48	41.86	4.36
